# Restricted posture in dentistry – a kinematic analysis of orthodontists

**DOI:** 10.1186/s12891-017-1629-7

**Published:** 2017-06-23

**Authors:** Daniela Ohlendorf, Christina Erbe, Imke Hauck, Jennifer Nowak, Ingo Hermanns, Dirk Ditchen, Rolf Ellegast, David A. Groneberg

**Affiliations:** 1 Institute of Occupational Medicine, Social Medicine and Environmental Medicine, Goethe-University, Theodor-Stern-Kai 7, 60590 Frankfurt am Main, Germany; 2 School of Dentistry, Department of Orthodontics, University Medical Centre of the Johannes Gutenberg University Mainz, Augustusplatz 2, 55131 Mainz, Germany; 3 Institute for Occupational Health and Safety (IFA) of the German Social Accident Insurance (DGUV), Alte Herrstraße 111, 53757 Sankt Augustin, Germany

**Keywords:** Restricted posture, Orthodontist, Cuela, Kinematic analysis

## Abstract

**Background:**

This study aims at identifying orthodontic activities with the highest frequency of unfavorable/awkward and static postures held over a period of more than 4 s based on kinematic analysis. Moreover, a separate analysis of static postures for orthodontic and non-orthodontic activities serves to evaluate the duration for which these particular postures are assumed.

**Methods:**

In total, 21 (13f/8 m) orthodontists (age: 31.5 ± 3.8 years) participated in this study. CUELA, a personal measurement system, was used to collect kinematic data for all orthodontic activities in a working day. Angle values of the head and torso were evaluated in accordance with ergonomic standards. Only those postures that were held statically for 4 s and longer were selected for further analysis. Alongside the kinematic analysis, the activities performed on-site were also subject to a detailed computerized analysis. The synchronization of data collected from both measurements arranges the patterns of posture found chronologically and in conjunction with the orthodontic activities performed ((I) “treatment” (II) “office” and (III) “other activities”).

**Results:**

For (I) we observed an anterior inclination of the head and torso area as well as a twist of the head and neck area to the right. We found anterior back inclination and lateral back torsion to the right for (II) and (III). If, furthermore, we differentiate the duration of static postures, there are primarily short to medium-term (4–30s) static postures identified for (I). Also, categories (II) and (III) predominantly demonstrate static back postures with a duration of up to 30 s. With regard to (II) we observed that the back is ventrally inclined for 10.1% of the total activity duration.

**Conclusions:**

During treatment static strains are observed in the entire head and torso area. On the contrary, static postures prevalent in the torso area are essential for activities of the other categories, particularly office work. These findings allow for a careful selection of unfavorable and static postures for each of the activities performed and help to develop specific preventive measures.

## Background

Studies around the globe point to the high prevalence of musculoskeletal disorders in orthodontists regardless of their given work experience [1–8]. These studies show an increased pain symptomatology especially in the neck, shoulder, and/or back area caused by dental activities [9–15]. Moreover, Blanc et al. [16] found out that at different dental treatment units muscle activities and the joint angles assumed vary with the type of occupational posture. As a result, there is evidence that musculoskeletal disorders and the pain caused often constrain the work of dentists and orthodontists [1, 17, 2] or even force them to retire early on grounds of occupational disability [18, 19]. Muscular dysbalances and the resulting disorders develop primarily due to poor occupational posture [12, 20, 21]. These disorders most likely originate in working continuously in static postures but also in often-repeated workflows [22, 17, 23]. Hereby, static refers to maintaining an unfavorable (restricted) posture which requires more static muscle activity and possibly results in excessive muscular strain as a consequence [24].

Various ergonomic concepts were developed to decrease musculoskeletal disorders. As early as 1972, Schön [25] discovered that static muscular fatigue occurs during dental activities but also stated that contrary to standing muscular fatigue decreases in a sitting position by 40% despite the increased pressure on the intervertebral discs. With ventral inflexion and rotation of the torso, a posture often assumed by dentists, the pressure on the spinal disc even increases by 400% [25]. Disorders caused by spinal disc issues can result in occupational disability [26–28].

Rohmert et al. [15] conclude that a change in posture to relax muscles is key to prevent signs of fatigue in the various muscle groups. An ideal approach to inhibit fatigue in the muscle groups is an exposition of 30 s followed by a micropause of a few seconds. In occupational medicine static posture is defined as any posture held for more than 4 s [29, 30].

According to the surveys by Valachi et al. [26, 28] continuous static postures that involve more than 50% of the body muscles for stabilization present one of the main causes for musculoskeletal disorders in dentists and are thus considered more harmful to the human body than dynamic activities [26]. Previous studies used RULA (rapid upper limb assessment), for instance, to measure static postures in dentists [31, 32]. RULA serves to evaluate risk factors for job-related musculoskeletal disorders in the upper limbs. This method thereby measures static postures for a duration of more than one minute [31]. Park et al. [32] have demonstrated with RULA that the risk for dentists of developing musculoskeletal disorders is the highest in the low back and neck. Moreover, they found that the posture of dentists routinely comprises neck rotation, shoulder abduction, as well as a strong inclination of the torso to front [33].

Although, the routine of orthodontists differs greatly from the routine of dentists, only a few studies have been conducted to date to measure occupational posture in orthodontists [34]. Therefore, our objective is to study the orthodontic workday through kinematic analysis following the RULA method. The continued measurement of postures and joint angles allows for a concise quantification of occupational postures as restricted postures. This analysis also aims to reveal that the overall percentage of non-neutral postures is higher in the torso than in the head and neck area for all three categories ((I) “treatment”, (II) “office” and (III) “other activities”). Moreover, the duration of static postures involved in the most common activities is also determined. Of particular interest here is whether the percentage of static postures held for more than 4 s is higher for treatment activities as opposed to office work by default.

## Methods

### Subjects

Twenty one (13f/8 m) orthodontic postgraduate residents employed at dental schools in Germany participated in this study. The average age of subjects was 31.5 ± 3.8 years and their work experience accounted for 3.9 ± 2.5 years. One dropout was recorded for the group of male participants. Among others, exclusion criteria for participants were signs of functional impairments of the musculoskeletal system due to spinal fusion or severe deformities of the spine (e.g.: scoliosis). Furthermore, injuries of the musculoskeletal system such as disc herniation and fractures in the back and neck, as well as muscular diseases that occurred more than 2 years prior to the study were criteria for exclusion.

Each participant was measured on a randomly selected workday. This study was approved by the Ethics Committee (135/14) of Goethe University in Frankfurt am Main. All participants signed an informed consent to take part in the study.

### CUELA measuring system

The CUELA system (computer-assisted acquisition and long-term analysis of stresses on the musculoskeletal system) was used to record the subjects’ body postures [35, 36]. CUELA is a personal system developed at the Institute for Occupational Safety and Health of the German Social Accident Insurance (IFA; Sankt Augustin/Germany) that uses sensors (accelerometers [ADXL 103/203] and gyroscopes [muRata ENC-03R] for head, arms, legs, back, potentiometers [Contelect] for back torsion) to measure the position or angle, and, in this way, allows for a kinematic reconstruction of the subjects’ motions. CUELA detected the probable degrees of freedom essential for a realistic description of dynamic motions at a sampling frequency of 50 Hz and with an angular accuracy of ±1°. Please refer to Table 1 for all calculated angle values [37–39].

**Table 1:** Depiction of the recorded body/joint angles based on DIN-EN 1005–4, applied evaluation parameters and assessment criteria according to ergonomic layouts

Body areas	Joint/Body area	Degree of freedom according to medical Definitions (posture concept)	Angle range according to ergonomic standards
Head/neck	Head	flexion/extension (H_f) (44)	Neutral: 0 to 25° Moderate: 25to 85° Awkward: < 0° & > 85°
		lateral inclination (H_li) (44)	Neutral: −10 to 10° Awkward: < −10° & >10°
	Cervical spine (CS)	flexion/extension (CS_f) (44)	Neutral: 0 to 25° Awkward: < 0° & > 25°
		lateral flexion (CS_lf) (44)	Neutral: −10 to 10° Awkward: < −10° & >10°
Back	Thoracic spine (TS)	flexion/extension (TS_f) (44)	Neutral: 0 to 20° Moderate: 20 to 60° Awkward: < 0° & > 60°
		lateral flexion (TS_lf) (44)	Neutral: −10 to 10° Moderate: −10 to −20° Moderate: 10 to 20° Awkward: < −20° & > 20
	Lumbar spine (LS)	flexion/extension (LS_f)	No ergonomic layout available
		lateral flexion (LS_lf)	
	Torso (T)	flexion/extension (T_f) (44)	Neutral: 0 to 20° Moderate: 20 to 40° Awkward: < 0° & > 40°
		Inclination (T_i) (44)	Neutral: 0 to 20° Moderate: 20 to 60° Awkward: < 0°& > 60°
		Lateral inclination (T_li) (44)	Neutral: −10 to 10° Moderate: −10 to −20° Moderate: 10 to 20° Awkward: < −20° & > 20°
		torsion (T_t) (44)	

### Measuring system: Objective work activity analysis with mini-PC

Observers use software specifically developed for work activity analysis to document the workflows of orthodontists in real time by the second on a portable hand-held computer (UMPC Samsung Q1, Samsung Electronics GmbH. Schwalbach, Germany). On the one hand, this approach will allow for identifying the particular work activity, whereas, on the other hand, the software can also determine the duration of these activities within these workflows. For a more detailed description of the system please refer the methods paper by Mache et al. [40, 41].

### Experimental design

**Fig. 1:**
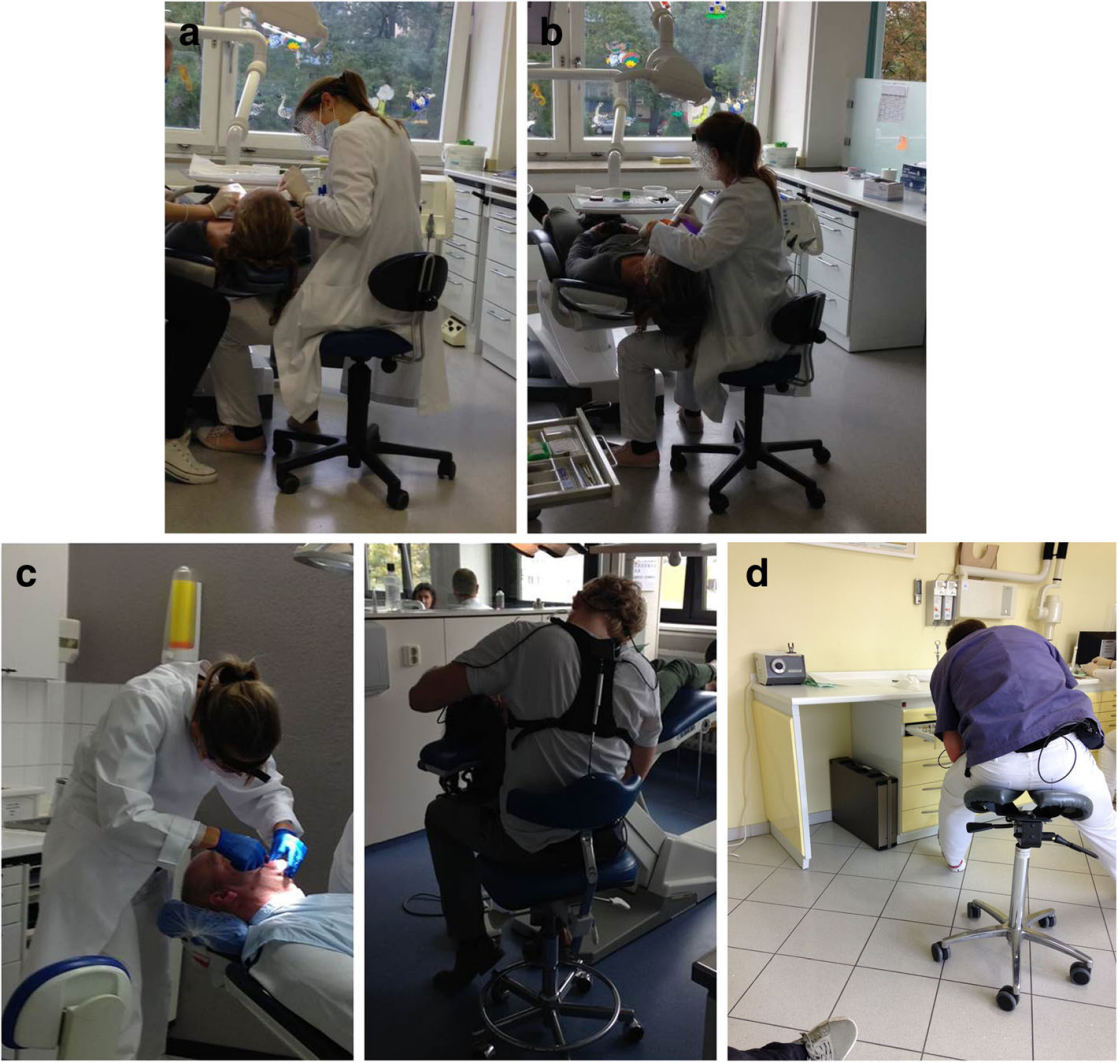
**a** Category I: Carry out craft activities. **b** Category I: Bonding of fixed appliances. **c**: Category I: Medical examination on a patient. **d**: Category III: Taking instruments

Prior to the study, the software was programmed in accordance with the work activity spectrum of orthodontists. All activities were divided into the categories (I) “treatment,” (II) “office,” and (III) “other activities.” These categories serve to describe all activities involved in the day-to-day routine of orthodontists (Table 2). The following figures illustrate examples of assigned activities of categories I and III (Fig. 1).

**Table 2:** Depiction of all categories with the respective work stages, their definition and the respective duration

Category	Sub-activities	Definition
Treatment	impressions	Taking an impression of the patient (teeth)
	consultation	Case discussion of two doctors on patients
	Mini Implant	Insertion of a mini implant
	archwire/elastic change	Replacement of archwire/elastics
	photo	Case documentation with the camera
	craft activities	Generic term for operations that do not fall into the above activities
	removable appliance	Insertion/control of removable appliances
	conservative dentistry	Cosmetic restorative filling (dental work)
	fixed appliance	Bonding/separation/repair of fixed appliances (mostly multibracket appliance)
	palpation	Palpation of the muscles of the face/temporomandibular joints of the patient
	break	Short breaks during treatment
	prophylaxis	Cleaning of teeth and brushing training
	splint	Insertion/control of occlusal appliance (splint)
	medical examination	First time/control examination of a patient
	angle piece/ultrasound	Usage of angle piece/ultrasound handpiece during the treatment
Office	file	Inspection of records (Findings/Dental Model/X-ray image)
	Office work	Data entry into patient record/PC Work
	model analysis	Analysis and design of the treatment plan using dental casts/X-ray images
	phone call	Phone conversations
Other activities	meeting	Medical meetings
	conversation	Discussions with patients and staff as solitary activities
	hygiene	Hygiene (washing/disinfecting hands, putting on gloves/mouth protection)
	Taking up/putting down of instruments	Take instruments from a drawer/store within and also before/after treatment
	laboratory	Any laboratory work
	walk	Covering distances

### Evaluation

Synchronizing the work activity analysis with the CUELA measurement in the CUELA software (IFA; Sankt Augustin/Germany) enables a temporal allocation of the motion patterns found and the associated activities. Relevance and duration of each work category were divided according to their percentage values. Angle values for each anatomical area (evaluation parameter) were then evaluated in compliance with ergonomic standards and assigned to a color-coded angle range representing ergonomic standards (traffic light: system red/yellow/green). Based on the respective colors postures are assessed as unfavorable, moderate (acceptable with reservations), or neutral [30, 42, 43] (Table 1). In reference with the criteria for classification the percentage for each evaluation parameter is calculated and assessed with regard to whether activities are executed in neutral, moderate, or unfavorable postures for all activities (categories I, II, and III). Then, the percentage of moderate and unfavorable postures are added up and presented in summary as values that represent non-neutral postures.

The overall statistics show the percentage of static non-neutral postures for the respective activities and for each evaluation parameter. Static postures are those postures that are ranked as moderate or unfavorable according to ergonomic standards as outlined by ISO standards [30] and held for more than 4 s. As postures can be held for a longer duration, the RULA method (rapid upper limb assessment) [31] has been used to apply a posture related screening method for static postures among dentists for durations of more than 1 min without further differentiation.

Based on these valuation methods for static postures we also distinguish in addition to the ≥4 s threshold between postures that are held for more than 60 s, between 30 and 60 s, and postures that are held between 4 and 10 s (statics components) [29, 30]. Furthermore, we calculate the quotient based on the total percentage of static and non-neutral postures to determine the percentage of static postures involved in non-neutral postures (total percentage of static non-neutral postures).

## Results

We were able to use a total of 95.9 h (5752.6 min) of data material excluding neutral postures and non-related activities such as breaks or toileting. Category I account for 34% (1952.9 min), category II “office” for 33% (1893.7 min) and category III “other activities” for 33%, (1906.3 min) of the total data material.

With regard to treatment (I) the study focused on the most frequently executed activities such as “craft activities,” “archwire/elastic change,” “contra-angle/ultrasound,” “medical examination,” “fixed appliance” and “removable appliance.” Of these six sub-activities “archwire/elastic change” accounts for 705.3 min (36%), “craft activities” for 408 min (21%), and “fixed appliance” for 325 min (17%) 74% of the total treatment time.

The analysis of kinematic data mainly focuses on non-neutral motions with a percentage of ≥50% of the total activity duration and a conspicuous percentage of static postures of ≥25%. If there were no anomalies found for the total percentage of static postures, data material for non-neutral postures of ≥75% was analyzed. These threshold areas were determined in relation to priority rankings.

Tables 3 and 5 show the percentage of postures for activities of the categories (I), (II), and (III) divided into ergonomic classifications (neutral, moderate, unfavorable). Moreover, these tables list the sum values derived from adding results for moderate and unfavorable postures as non-neutral postures. Tables 4 and 6 refer to the total percentage of static postures, the temporal differentiation, as well as the total percentage of static non-neutral postures.

**Table 3:**
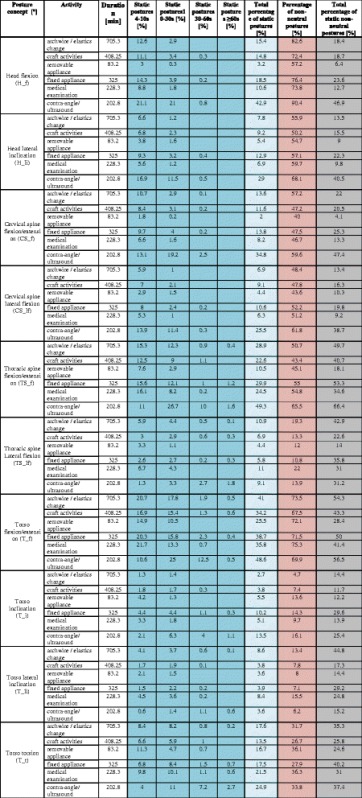
Treatment: Percentage of neutral, moderate, and unfavorable/awkward postures (%) of the total activity duration for the head and neck area as well as the percentage of non-neutral postures as the sum of all moderate and unfavorable/awkward postures (%). See Table 1 for color-coded ranking system

**Table 4:**
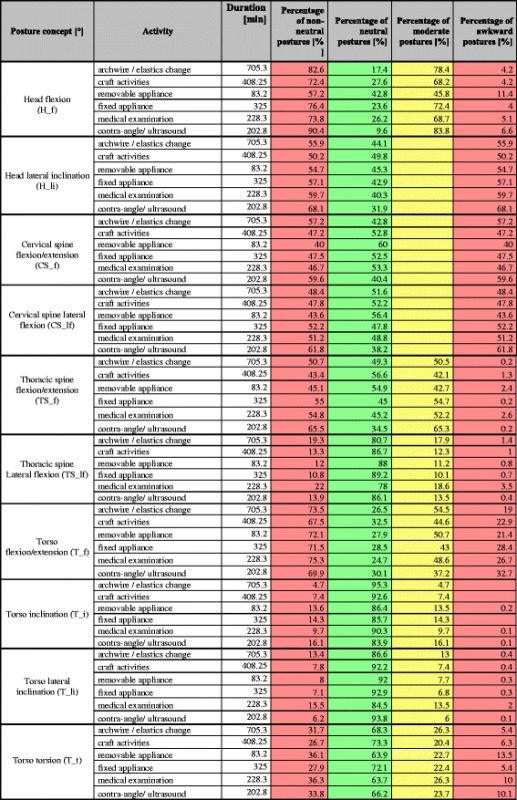
Percentage of static postures ≥4 s during treatment. Figure legend: total percentage of static postures = sum of all moderate and unfavorable/awkward postures that occur with all activities

### Category I: Treatment

In the head and neck area the percentage of non-neutral postures with head flexio/extension (H_f) during the activities “archwire/elastic change” and “craft activities” is at 82.6% or 76.4%, from which 15.4% or 18.5% are performed statically. The percentage of static postures for both activities is between 4 and 10 s at 12.6% and 11.1% and thus accounts for the largest share in this category. 90.4% of postures in the non-neutral range observed during the activity “contra-angle/ultrasound” demonstrated a percentage of static postures of 46.9%. Regarding static postures (42.9%) 21.1% are held between 4 and 10 s and 21% are held for 10–30 s.

During the activity “contra-angle/ultrasound” lateral inclination of the head (H_li) demonstrates a percentage of non-neutral postures at 68.1% with an overall percentage of static postures of 40.5%. Hereby, postures that last between 4 and 10 s account for 16.9%, and thus the largest percentage of static postures, followed by 11.5% of postures that last for 10–30 s.

For the same activity we observed conspicuous values with regard to the extension/flexion of the cervical spine (CS_f) and the lateral flexion (H_li). With cervical flexion/extension (CS_f) the percentage of non-neutral postures is at 59.6% with a total percentage of static postures of 47.4%.

With regard to the total percentage of static postures of 34.8% we find that 13.1% of postures are held between 4 and 10 s, 19.2% between 10 and 30 s, and 2.5% last between 30 and 60 s. The percentage of non-neutral postures for cervical spine lateral flexion (CS_lf) is 61.8% and accounts for 38.7% of the total percentage of static postures. The duration of static postures lies between 4 and 10 s (13.9%) and between 10 and 30 s (11.4%).

In the back area we found conspicuous results for flexion/extension of the thoracic spine (TS_f) regarding the sub-activities “archwire/elastic change,” “medical examination,” and “fixed appliance.” During “archwire/elastic change” and “fixed appliance” the percentage of non-neutral postures is 50.7% or 55%, which accounts for 49.7% or 53.3% of the overall percentage of non-neutral postures. 15.3% or 15.6% of static postures last between 4 and 10 s while 12.3% or 12.1% have a duration of 10–30 s. The percentage of non-neutral postures for “medical examination” is 54.8% and comprises 34.6% of the total percentage of static postures. Considering the overall allocation of static postures (24.5%), the percentage of static postures that are held between 4 and 10 s accounts with 16.1% for the largest share, followed by 8.2% of static postures that are held for 10–30 s.

With torso flexion/extension (T_f) we found conspicuous values regarding the percentage of non-neutral postures for all activities performed (67.5% – 75.3%) as well as a conspicuous total percentage of static postures between 28.5% – 56.5%. 10.6% – 21.7% of static postures have a duration of 4–10 s, 10.5% – 25% last between 10 and 30 s, 0% – 12.5% are held between 30 and 60 s, and 0% – 0.6% are maintained for 60 s and longer (Table 3, Table 4).

### Categories II and III: Office and other activities

Static postures in category II (“office”) were analyzed based on the sub-activity “office work” whereas static postures in category III (“other activities”) were analyzed in relation to the sub-activities “conversation” and “taking up/putting down of instruments.”

In the head and neck area “office work” (category II) is executed in non-neutral postures with extension/flexion of the cervical spine (CS_f) 57.3% of the time. This accounts for 32.9% of the total percentage of static non-neutral postures. The duration of static postures is primarily between 4 and 10 s (9.1%) and 10–30 s (8.1%).

Seventy five percent of postures with extension/flexion of the Torso (T_f) are in the non-neutral range, from which 54.8% are executed statically. Based on these results we calculated a total percentage of static postures of 66%. Moreover, we found that 10.1% of static postures are held for 60 s or longer, 12.3% between 30 and 60 s, 20.5% between 10 and 30 s, and 11.9% have a duration between 4 and 10 s (Table 5, Table 6).

**Table 5:**
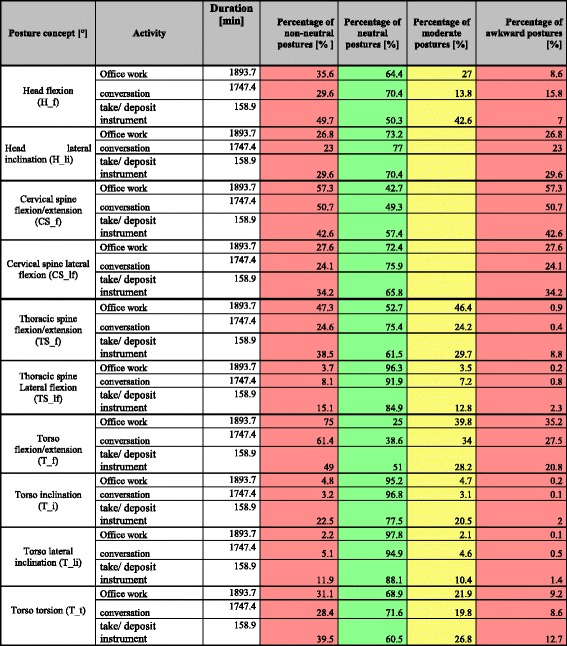
Office and other activities: treatment: percentage of neutral, moderate, and awkward postures (%) of the total duration in the head and neck area as well as the percentage of non-neutral postures as the sum of all moderate and awkward postures (%). See Table 1 for color-coded ranking system

**Table 6:**
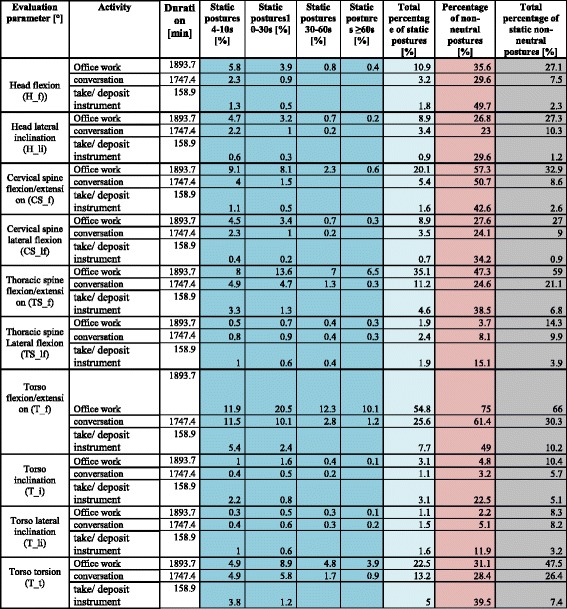
Percentage of static postures held for ≥4 during office work and other activities. Figure legend: Total percentage of static postures = Sum of all moderate and awkward postures that occur with all activities

With regard to category III (other activities) the percentage of non-neutral postures in the head and neck is <50% with a conspicuous total percentage of static postures of <25% as well as a total percentage of non-neutral postures of <75%.

The sub-activity “conversation” generated conspicuous results in the extension/flexion of the torso. From a total percentage of static postures of 30.3%, participants assumed non-neutral postures 61.4% of the time. 11.5% of static postures involved in the sub-activity “conversation” had a duration between 4 and 10 s, 10.1% between 10 and 30 s, 2.8% between 30 and 60 s, and 1.2% had a duration of 60 s or longer (Table 5, Table 6).

## Discussion

Particular motion patterns are executed repeatedly during daily workflows of orthodontists [34]. These motion patterns can be short-term as well as long-term, dynamic or static. Musculoskeletal disorders in dental professions often originate in static positions that mostly comprise unergonomic sitting postures held incessantly during treatment as well as periodically repeated motions [22, 23, 17].

With frequently assumed treatment positions the practitioner’s body tilts forward whereby the head, neck and torso rotate laterally to gain the best possible view of the inside of patient’s mouth. For right-handed subjects, this result in a head rotation to the left and a head flexion to the right [26].

Within this treatment position, data generated in the present study affirms that static postures (4–10 s) in the head and neck area have a shorter duration than static postures in the back, even though we found that in both anatomical areas positions assumed were primarily inclined to the front. Static postures in the back area last between 4 and 10 s and are almost as frequent as postures that are held between 10 and 30 s. If static postures are assumed for more than 60 s, they primarily refer to restricted and laterally inclined postures of the torso during the sub-activity “contra-angle/ultrasound” (inclination of the thoracic spine to the right (TS_lf), inclination of the torso to the right (TS_lf), back torsion to the right (T_r)). At the same time, we also found a high percentage of static postures of 46.9% for moderate and unfavorable postures.

As a result, head and neck postures are adjusted in shorter intervals than back postures. The risk of developing work related musculoskeletal disorders is particularly high in the back and neck, a conclusion that is also confirmed by Park et al. [32] and their application of the RULA method.

The back curvature demonstrates similarly high percentage static postures values for activities in category II and III. Nevertheless, the risk that tilted positions bear is smaller with office work as it is an activity executed in supported positions that decrease static muscle strain.

The evaluation of our data on static postures clearly shows that orthodontists remain, especially in the back area, in static anterior inclined postures due to long hours of executing office work and orthodontic treatment. However, ranking static postures, the total duration of the respective activity as well as the percentage of the individual statics components shall be considered. Therefore, we selected only those activities which are the most significant for the orthodontic workflow with regard to duration and frequency (Table 3; Table 5).

The orthodontic treatment of patients is usually an unsupported activity, which results in greater muscular strain. Moreover, it should be noted that particularly results for “office work” can be inaccurate as the measurements conducted could not generate data on support provided by the back of the chair or a wall, for instance. Additional video recording could be used to evaluate the results more profoundly. However, ethical concerns regarding the patient’s right to privacy and confidentiality might counter argue the use of video recording as not every orthodontic patient wants to be filmed during treatment.

A Scandinavian study Keruso et al. [44] affirms the most common musculoskeletal disorders among orthodontists. Dentists, orthodontists, and office clerks (control group) were surveyed on health issues and the results of 70% to 72% demonstrate that the differences between dentists and orthodontists are rather minor. However, office clerks appear to encounter significantly less musculoskeletal pain as dentists and orthodontists combined.

Despite the similar field of patient treatment, orthodontic activities (check-ups, archwire change, or rebonding brackets) are not identical with general dental activities (restorative fillings, impressions, preparation of dentures, teeth extraction). Although, orthodontists handle more office work than dentists (ORTHO: treatment 34% vs. office 33%; DDS: 41% vs. 23%), which renders a large share of their treatment theoretical work (model analysis and concise planning of treatment process), they nonetheless execute many activities in anterior inclined and static postures [34]. As a result, orthodontists carry a greater risk of developing work related musculoskeletal disorders due to excessive static stress. Moreover, existing issues with the musculoskeletal system are often related to prolonged static positions [17, 22, 23, 45]. As early as 1972, Schön [25] observed muscular strain in static postures. According to Valachi et al. [26] frequently assumed static positions are more harmful to the human body than dynamic activities. Furthermore, the authors of this study also found that participants assumed static postures more often than dynamic postures. Thereby, more than 50% of muscles are required to hold a motionless position, which results in fatigue and, with frequent repetition, also in pain. In our opinion, the conclusion of Valachi et al. [28]- is also valid for both, dentists and orthodontists, as their positions during treatment are similar [34].

The kinematic analysis measures the total duration and frequency of static postures conducted by all participants. However, the analysis does not take pauses between the same activities into account. As mentioned earlier, the present data material does not serve to distinguish between supported and unsupported postures. Also, to date no kinematic analysis of fine motor movements in the fingers, hands, and wrists has been conducted yet (25% or 44% suffer from pain in their hands), even though these movements are essential for dental professionals executing concise and delicate tasks [46, 10].

## Conclusions

The kinematic analysis of head and torso postures shows a prevalence of static postures in orthodontists. Head and neck postures are adjusted in shorter intervals than back postures. The risk of developing work related musculoskeletal disorders is particularly high in the back, neck and head region. Since most positions of orthodontists during a working day were primarily inclined to the front, i.e. using a “contra-angle” or “ultrasound”. Moreover, the study emphasizes that postures ranked as moderate (according to ergonomic norms) paired with static strain can result in restricted postures. These postures present health risks in the workplace as they increase the probability of developing musculoskeletal disorders and are associated with activities on the job. The results of this analysis paired with the ergonomic classification of joint angles into the same categories and activities allow for a quantitative evaluation of the orthodontic profession in relation to the physical strains and the health risks for the musculoskeletal system.

## Abbreviations

CUELA-System: Computer-assisted acquisition and long-term analysis of musculoskeletal loads
RULA: Rapid upper limb assessment
